# Design and Measurement Properties of the Online Gambling Disorder Questionnaire (OGD-Q) in Spanish Adolescents

**DOI:** 10.3390/jcm9010120

**Published:** 2020-01-02

**Authors:** Joaquín González-Cabrera, Juan M. Machimbarrena, Marta Beranuy, Priscila Pérez-Rodríguez, Liria Fernández-González, Esther Calvete

**Affiliations:** 1 Faculty of Education, Universidad Internacional de la Rioja (UNIR), Avenida de la Paz, 137, 26006 Logroño, Spain; 2 Faculty of Psychology, Universidad del País Vasco (UPV/EHU), Avenida de Tolosa, 70, 20018 Donostia, Spain; 3 Faculty of Psychology and Education, University of Deusto, Avenida de las Universidades 24, 48007 Bilbao, Spain

**Keywords:** online gambling disorder, pathological online gambling, Internet, adolescents, questionnaire, validation

## Abstract

Gambling disorder is of great clinical and social relevance since it seriously affects people who suffer from it. More recently, the Internet has exacerbated the problem with online casinos, poker, and sports betting. However, there is little evidence of this problem, and we know of no diagnostic questionnaire. The main objectives of this study were to develop the Online Gambling Disorder Questionnaire (OGD-Q) for adolescents, evaluate its main psychometric properties, and establish diagnostic criteria to differentiate pathological from non-pathological online gamblers. We conducted a study in 16 schools across seven regions of Spain, sampling 2691 adolescents, 883 of whom had reported some online gambling experience. Of those, 602 were boys (68.2%) and 281 were girls (31.8%) Sampling was non-probabilistic and incidental. Mean age and standard deviation were 14.25 ± 1.55 (11–19 years). Confirmatory factor analysis yielded a one-dimensional model with a good fit. The reliability indicators were satisfactory (>0.94). The scores on the OGD-Q were related to other constructs, such as Internet gaming disorder, problematic Internet use, and nomophobia. Participants classified as having problems or being at risk of online gambling disorder presented significantly more stress, anxiety, and depression. Participants categorized as having online gambling disorder comprised 0.89% (*n* = 24) of the total sample and 2.71% of those who have gambled at some time. We discuss these findings and their practical implications in this article and propose future lines of research.

## 1. Introduction

Access to the Internet has expanded in recent years, thanks to smartphones. More than 83% of Spanish households have access to the Internet through optical fiber or ADSL (Asymmetric Digital Subscriber Line) connections, and in parallel, 97% of the population has mobile phones that can connect to the Internet [1]. The National Statistics Institute [2] reveals that, at the age of 10, more than 85% of children have access to the Internet, and by the age of 12, more than 75% have their own cell phone. By age 14, that number increases to more than 90%. The Internet has led to remarkable progress in education, healthcare, and communication, as well as contributions to our leisure and economic lives, but it also involves risks such as cyberbullying, grooming, sexting, or cyberdating abuse [3]. These risks are especially worrisome in adolescence, a time of emotional, social, and cognitive development [4]. In addition, new generations (especially the so-called post-millennials or Generation Z) can conflate offline and online reality [5]. Continuous Internet connection contributes to the generation gap as well as to new psychosocial issues [6,7]

Many traditional psychosocial problems have migrated online in recent years. For instance, bullying has given way to cyberbullying [8], grooming to online grooming [9], and intimate partner violence to cyberdating abuse [10]. New problems have also emerged, such as problematic Internet use [11], nomophobia [12], and online video-game disorder [13]. The latter may result in problematic use of the Internet, which can lead to dependency or addiction, even without the use of chemical substances [14]. Addiction and dependence detract from individuals’ freedom, limiting their awareness and restricting their interests [15]. Since its fifth edition, the Diagnostic and Statistical Manual of Mental Disorders (DSM-5) [16] has included addictive behaviors, such as gambling disorder (312.31) [17]. In the International Classification of Diseases (ICD-11) [18], pathological gambling is presented under heading 6C50 (Gambling Disorder), although there has been a notable advance with respect to the DSM-5 by adding the nuance of “predominant online gambling” (6C50.1). Additionally, some symptoms play a key role in the transition from healthy gambling to pathological gambling (either online or offline). They are the same ones that characterize psychological dependence: craving, polarization or attentional focus, mood modification, and loss of control or sense of helplessness [19,20].

A body of evidence suggests that Internet gambling is more addictive than traditional gambling, [21,22], especially for problematic gamblers [23,24], possibly due to the variety, design, and marketing of games.

Gambling is prevalent among adolescents and young university students between the ages of 16 and 24 [25], when they are most vulnerable to developing emotional, cognitive, and behavioral problems related to online gambling [25,26,27]. Currently, gambling and other associated pathologies are more common in males than in females [25,26], though there has been a significant increase in online gambling among women recently [28].

Notably, gambling disorder is associated with numerous other mental health issues, such as depression and the use of addictive substances—mainly alcohol [24,29,30]—as well as other addictive behaviors, such as problematic Internet use [31,32]. In addition, it has been linked to borderline and narcissistic personality problems [33], symptoms of stress and anxiety [34], as well as cognitive distortions, such as the illusion of control [35].

So far, there does not seem to be a specific diagnostic tool for online gambling disorder in adolescents. The most similar measuring instrument is the Online Gambling Symptom Assessment Scale (OGSAS) of Kalkan and Griffiths [36], which is intended to assess the consequences of problematic online gambling. The OGSAS is an adaptation of the Gambling Symptom Assessment Scale (G-SAS) [37] for online gambling disorders.

This background shows a pressing need for tools to help diagnose and evaluate online gambling disorder. Thus, the main objectives of this study were to develop the Online Gambling Disorder Questionnaire (OGD-Q) for adolescents, evaluate its main psychometric properties, and establish diagnostic criteria to differentiate online gambling disorder from non-pathological online gambling disorder. To establish convergent validity indicators, we studied the associations between OGD-Q scores and problematic and generalized Internet use, nomophobia, Internet Gaming Disorder, and symptoms of anxiety, depression, and stress.

## 2. Experimental Section

### 2.1. Design and Participants

We conducted our cross-sectional study in March and April 2019. The initial number of participants was 2691 students of whom 1268 were boys (47.1%) and 1423 were girls (52.9%) from 16 Secondary Compulsory Education (SCE) schools in 7 Spanish regions: northern Spain—Asturias (*n* = 838, 31.14%), Castilla León (*n* = 558, 20.74%), and the Basque Country (*n* = 192, 7.13%); eastern Spain—Aragón (*n* = 142, 5.28%) and Valencian Community (*n* = 438, 16.28%); and central Spain—Madrid (*n* = 221, 8.21%) and Castilla la Mancha (*n* = 292, 10.85%). Ten participants did not indicate their SCE. The final sample was composed of the participants (*n* = 883) who reported having had some experience in online gambling (602 boys—68.2%—and 281 girls—31.8%). The mean and standard deviation of ages was 14.25 ± 1.55, with a range between 11 and 19 years. The distribution of students by cycles and school grades was as follows: 199 students from the 1st grade of SCE (132 boys and 67 girls); 185 students from the 2nd grade of SCE (114 boys and 71 girls); 185 students from the 3rd grade of SCE (133 boys and 52 girls); 227 students from the 4th grade of SCE (170 boys and 57 girls); 47 students from the 1st grade of high school (Bachillerato) (32 boys and 15 girls); and 40 students from the 2nd grade of high school (Bachillerato) (21 boys and 19 girls). Sampling was non-probabilistic and incidental.

### 2.2. Instruments

The participants provided information about demographic variables such as sex, grade, school, and age. The following instruments were used to analyze the variables under study. The items referred to the time elapsed since the start of the school year (approximately five months).

The Online Gambling Diagnostic Questionnaire (OGD-Q) was designed according to national and international guidelines and standards for the design of questionnaires (American Educational Research Association, American Psychological Association, and National Council on Measurement in Education [38], as described by Muñiz and Fonseca-Pedrero [39]).

We followed this procedure: (1) We defined the measurement variable and tool specifications (for example, application requirements, type and number of items and questionnaire length, content and distribution of the items, specifications, and instructions). (2) We created a table including the questionnaire’s specifications, indicating which construct would be evaluated, its items, and the differential importance of each item. (3) We conducted a pilot run of the tool. (4) Before the final administration, we conducted two cognitive interviews with two adolescents to analyze the psychological comprehension of the items [39].

The questionnaire was designed by adapting the criteria for the traditional gambling disorder of the DSM-5 (312.31) to the online context, the ICD-11 criteria to predominantly online gambling disorder (6C50.1), and the recommendations of numerous experts [15,19,20]. We noted that online gambling disorder could be diagnosed in an individual who shows persistent and maladaptive online gambling behavior that includes at least four or more of the criteria presented in Table 1 and that are linked, among others, to salience, euphoria, tolerance, withdrawal, conflict, and relapse or craving. Each indicator was transformed into an item (see Table 1 for items in English and Supplementary Table S1 for items and instructions in Spanish). We then used a five-point Likert-type response scale that ranged from 1 (never) to 5 (every day) to measure the frequency of gambling. The total OGD-Q scores ranged between 11 and 55 points. In addition, those who reported at least one behavior had to indicate how long they had been performing it. The response options were: recently (for less than a month), for more than a month, for more than six months, and for more than 12 months.

Three experts in the area of study determined the criteria using an online focus group, achieving high inter-judge reliability (>0.8) in the different elements and construct specifications [39]. After the focus group, we edited the questionnaire. We tested the final version in a pilot run with 33 participants, who were not included in the final sample. Preliminary results reflected adequate tool functioning at the psychometric level. Finally, the two cognitive interviews indicated an adequate comprehension of the items.

Spanish version of the Generalized and Problematic Internet Use Scale (GPIUS2) [11,40]. This scale consists of 15 items divided into five factors: (1) Preference for online social interaction; (2) Mood regulation; (3) Negative consequences; (4) Cognitive concern; and (5) Compulsive use. These last two dimensions are sometimes grouped into a single dimension: Poor self-regulation. Agreement with the items is rated on a six-point Likert scale ranging from 0 (completely disagree) to 5 (completely agree). The reliability obtained for this study can be seen in Table 3.

Internet Gaming Disorder Scale (IGD-20) [13,41]. The questionnaire consists of 20 items that evaluate online and offline video-game activity that has been performed over the past 12 months in terms of the diagnostic criteria for the internet gaming disorder ([IGD] [16]). At the same time, the 20 items draw on the theoretical model of addiction components established by Griffiths [20] due to the overlap between the nine IGD criteria and the addiction components model. Consequently, the IGD-20 evaluates six different dimensions: (1) Prominence; (2) Mood modification; (3) Tolerance; (4) Withdrawal symptoms; (5) Conflict; and (6) Relapse. It uses a five-point Likert scale, ranging from 1 (strongly disagree) to 5 (strongly agree). The reliability (Cronbach’s alpha and the omega coefficient) obtained for this study was α = 0.91 and ώ = 0.92, respectively. The reliability of each dimension in this study can be seen in Table 3.

Spanish version of the Nomophobia Questionnaire (NMP-Q) [7,12,42]. This tool evaluates four dimensions: (1) Not being able to communicate; (2) Loss of connection; (3) Not being able to access information; and (4) Giving up comfort. The response format uses a seven-point Likert scale, ranging from 1 (strongly disagree) to 7 (strongly agree). Scores range between 20 and 140 points. The reliability obtained for this study can be seen in Table 3.

Depression, Anxiety, Stress Scales-21 (DASS-21) [43,44]. This is a self-report that evaluates negative emotional states. It consists of 21 statements distributed in three subscales with seven items each: Depression, Anxiety, and Stress. It is rated on a four-point Likert scale ranging from 0 (does not apply to me) to 3 (it applies to me a lot or most of the time). The reliability of each dimension for this study can be seen in Table 4.

### 2.3. Ethical Considerations

The study was conducted with the informed consent of the participants and the school directors. The schools provided the parents or tutors with a consent form and informed them about the purpose of the study, its characteristics and promoters, and their right not to participate. Those parents/tutors who did not wish to allow participation returned the signed form indicating dissent. Less than 1% of the sampled participants refused to participate. The study was approved by the Research Ethics Committee of the International University of Rioja (PI:004/2019) and by the Research Ethics Committee of the Principality of Asturias (Ref. 231/17). There were no exclusion criteria, except for the refusal to participate by the legal guardians or by the students themselves.

### 2.4. Data Analysis

We carried out statistical analyses using the Statistical Package for the Social Sciences (v. 23.0.0) [45], the R software (v. 3.6.1) [46], the psych package (v. 1.8.12) [47], and the Lavaan package (v. 0.6-5) [48].

First, we analyzed the psychometric properties of each item, calculating the arithmetic mean, the standard deviation, the item-total correlation, the percentage of positive responses to each item, and the factorial loadings of each item (see Table 2). To meet our criteria, no item could fail to fulfill two of the following three statistical indices: (a) mean between 1.5 and 2.5, (b) standard deviation equal to or greater than 1, (c) item-total correlation equal to or greater than 0.35, and (d) skewness and kurtosis.

After verifying the assumptions (using the Kaiser-Meyer-Olkin index and Bartlett sphericity test), we examined the structure of the OGD-Q initially with an exploratory factor analysis (EFA) of its items. We used the principal components factor extraction method with Oblimin rotation. We then performed a confirmatory factorial analysis (CFA) using a Weighted Least Square Mean estimate. The imputation algorithm was employed to treat missing values, using the expectation-maximization method. The hypothesized model was one-dimensional, with the 11 items loading on the same factor. Following the recommendations of Hu and Bentler [49], we assessed goodness of fit with the Satorra-Bentler χ2 (S-Bχ2) index, the comparative fit index (CFI), the non-normative fit index (NNFI), the root mean square error of approximation (RMESA), and the standardized root mean square residual (SRMR). In general, CFI and NNFI values of 0.95 or higher reflect a good fit. RMSEA values of less than 0.06 indicate an excellent fit, whereas values between 0.06 and 0.08 indicate an acceptable fit. Finally, SRMR values lower than 0.08 indicate adequate fit.

To determine the internal consistency of the instruments employed, we estimated Cronbach alpha, ordinal, and omega coefficients [50].

In addition, we performed the following analyses: (a) analysis of frequencies and central trend and dispersion measurements of the study variables; (b) chi-square analysis for the contrast of proportions and analysis of adjusted standardized residuals; (c) *t*-test or Welch’s test for independent samples; (d) calculation of the effect size with Cohen’s d statistic when significant differences were found between two variables; (e) bivariate Pearson correlations; (f) analysis of variance with post-hoc Games–Howell comparisons. A value of less than *p* = 0.05 was considered significant.

To obtain the prevalence of online gambling disorder, all the items of the OGD-Q were dichotomized. Any item with a rating equal to or greater than 3 (frequently, very often, and every day) on the Likert scale was categorized as a “problem”. Items with scores below 3 (never and occasionally) were categorized as “no problem.” After this process, the scores obtained in all the items of the questionnaire were added. The criterion of “online gambling disorder” was established for those who obtained a score of 4 or more (meaning they had problems in four or more areas) over the past 12 months or more. This category is in line with the diagnosis of gambling disorder in the DSM-5 [16]. Participants who obtained four or more indicators over a period of 6 to 12 months were included in the “problem of online gambling” category. The category “at risk for online gambling problems” was used for those who scored on 4 or more indicators, but for less than the last 6 months.

## 3. Results

### 3.1. Factor Structure

The results of the EFA are as follows: The Kaiser-Meyer-Olkin index data and the Bartlett sphericity test obtained values of 0.951 and χ^2^ = 7182.532, *p* < 0.001. The inter-correlation matrix between the items was appropriate for the EFA. The results indicated a single factor that explained 68.6% of the total sample variance. A CFA was then carried out on a one-factor model, obtaining adequate fit indexes: S-Bχ^2^ (44, *n* = 883) = 87.523, *p* < 0.001; RMSEA = 0.010 (95% CI (0.000, 0.013)); CFI = 0.998; NNFI = 0.997, and SRMR = 0.037. Standardized factor loads (see Table 2) were high across all items, varying between 0.63 and 0.86. The Cronbach alpha and omega coefficients for the OGD-Q were 0.94 and 0.95, respectively.

### 3.2. Evidence of Validity of OGD-Q Scores

Table 2 presents other psychometric indicators for each of the OGD-Q items, namely, the mean, standard deviation, item-total correlation, and the percentage of positive responses to each item. At the psychometric level, the scores obtained for the items revealed problems in the mean and standard deviation of all items. However, the item-total correlations were satisfactory in all of them. Participants responded to between 8.9% and 16.7% of the items positively at least once. When we calculated skewness and kurtosis for each item, we found the values indicated a positively skewed distribution (see Table 2).

### 3.3. Differences by Sex and School Grade

The scores for all the items were higher for boys than for girls (*p* < 0.001), and the effect size was small in all cases (*d* < 0.3). The only significant differences among school grades appeared in Item 3 (*p* < 0.030), where students in the 1st grade of SCE scored higher than those in the 1st grade of high school, with a small effect size (*d* < 0.3).

### 3.4. Associations with Other Variables

To stablish convergent validity, we obtained correlations between the total score of the OGD-Q and the IGD-20 (*r* = 0.19, *p* < 0.001), the GPIUS2 (*r* = 0.06, *p* = 0.153), and the NMP-Q (*r* = 0.06, *p* = 0.206). The correlations between the OGD-Q and the DASS-21 were, for stress, depression, and anxiety, respectively, *r* = 0.13, *p* = 0.005; *r* = 0.159, *p* < 0.001; and *r* = 0.18, *p* < 0.001. There were also correlations between anxiety, stress, and depression and the IGD-20 (*r* = 0.25, *p* < 0.001; *r* = 0.21, *p* < 0.001; *r* = 0.20, *p* <0.001, respectively) and with nomophobia *r* = 0.44, *p* < 0.001; *r* = 0.39, *p* < 0.001; *r* = 0.48, *p* < 0.001, respectively). Table 3 and Table 4 show the correlations between the OGD-Q and the dimensions of each of the aforementioned questionnaires as a function of sex.

### 3.5. Prevalence of the Online Gambling Disorder

Higher scores on the OGD-Q indicated greater online gambling problems. The mean score and standard deviation for the study sample were 13.01 ± 5.43, with a range of 11–50. Following the DSM-V indications (4 or more criteria for 12 months or more), participants categorized as having “online gambling disorder” reached 0.89% (*n* = 24) of the total sample, and 2.71% of those who have gambled sometime. Those categorized as “problem with online gambling” (4 or more criteria between 6 and 12 months) represent 0.77% (*n* = 21) with regard to the total sample, and 2.38 compared to those who have ever gambled sometime (see Table 5). Those categorized as “at risk for problem online gambling” (4 or more criteria between recently and 6 months) made up 0.56% (*n* = 15) of the total sample and 1.7% of those who have ever gambled. Of the 60 adolescents who were problematic or at risk, 50 were boys and 10 were girls.

### 3.6. Psychological Distress of Participants with Online Gambling Disorder Problems

Table 6 shows the number of participants who presented four or more items/symptoms of psychological distress in the OGD-Q and those who had less than four items in the three dimensions of DASS-21. We noted significantly higher scores for depression, anxiety, and stress (with effect sizes greater than 0.68). The amount of time participants with four symptoms had been gambling online (12 months or more, between 6 and 12 months, less than 6 months) made no significant difference to their experience of stress, *F* (2, 47) = 0.338, *p* = 0.715; depression, *F* (2, 45) = 0.321, *p* = 0.727; or anxiety, *F* (2, 48) = 1.123, *p* = 0.324.

## 4. Discussion

### 4.1. Psychometric Properties of the OGD-Q

The OGD-Q is the first questionnaire that specifically aims to address the diagnosis of online gambling disorder. The questionnaire was developed with conceptual and methodological rigor as well as adequate criteria of validity and reliability to be used in the adolescent population in which the study was carried out. It is based on the diagnostic criteria for gambling disorder DSM-5 and the ICD-11, as well as criteria of significant authors in this field [14,15,19,20], adapting these criteria to adolescents in the online context.

Factor analysis confirmed a single factor with adequate goodness of fit, and there were also high item-total correlations and satisfactory factor loadings on all items. Reliability analyses indicated high internal consistency. However, the skewness and kurtosis values were not normal for any of the items. The OGD-Q has a positively skewed distribution because it is a diagnostic questionnaire, and most participants do not present the problem. We followed a procedure similar to one previously used to create questionnaires to determine inappropriate use of the Internet and smart phones, such as online video-game disorder [13,41] or nomophobia [7,12,42], among others. Hardly any research currently links online gambling disorder with other constructs, but there is evidence of a relationship between problematic use of the Internet and problems with online video games [51] and a relationship between nomophobia and problematic Internet use [12].

The results of our examination of the relationship between the OGD-Q and psychological problems such as stress, anxiety, and depression consistent with reports in other works [34,52,53].

We also found a relationship between online gambling and the dimension of negative consequences related to problematic Internet use. This finding has a theoretical-conceptual relationship with Caplan’s model [40], since online gambling disorder can affect social and personal functioning. It also coincides with Griffith’s model [20], which emphasizes the negative effects of the symptoms of addiction.

### 4.2. Prevalence of Online Gambling Disorder

Participants who scored four or more criteria in the last six months were classified as having a “problem with online gambling” and those who scored on four or more criteria in a period of less than six months were categorized as being “at risk”. Therefore, taking into account participants with problems and at-risk participants, the percentage of prevalence of problematic situations with online gambling would be just over 2% of the total sample and almost 7% of those who reported having some experience of online gambling.

These values are similar to those included in other studies on gambling. For example, Chóliz and Lamas [25] presented values above 7% for pathological gamblers, although they included both online gambling and traditional gambling. In Germany, Giralt et al. [54] presented values of between 1.7% and 2.2% for adolescent problem gamblers, also jointly considering online gambling and traditional gambling. In our sample, online gambling disorder was more prevalent in boys than in girls, as other studies have shown [25,26,28]. It should also be stated that the prevalence of this problem is very serious if we consider that children under 18 years of age cannot legally bet. Moreover, most studies combine online and traditional gambling, and the data in our study only report on the online version, so the underlying issue of gambling could be even greater. Becoña [55] already expressed this concern in his review of pathological offline gambling in adolescents and young people, which found a very high prevalence (between 1.7% and 8%). This data may increase over time, and each new study may find slight increases in the prevalence.

### 4.3. Practical Implications, Limitations, and Future Lines of Research

This study has several practical implications. It presents the design and validation of a tool with diagnostic criteria that can help to diagnose and analyze the prevalence of the problem. It makes sense of the important impact that this problem is beginning to have on a vulnerable population (adolescents) and also highlights the relationship of this problem to other Internet-related problems, as found in studies on other cyber-risks [3]. Knowledge of these data is of great interest to psychology and psychiatry units, as well as to school counselors and legal tutors. In view of its prevalence, prevention and intervention programs could address the new online facet of this problem. These could be integrated within a general framework of risk prevention in Internet use. In the future, discussing the inclusion of this disorder in the DSM might prompt further study and generate more evidence on this issue. The present study presents some limitations. Firstly, the OGD-Q is a self-reported questionnaire, so response and desirability biases are possible. Developing complementary measures, including the other-reports, could address this issue. Secondly, although we attempted to be rigorous and thorough by including different diagnostic manuals and authors, the validity of the evaluated content is exploratory and requires further revisions. Not having been able to carry out a test-retest of the questionnaire is also a limitation of the study. Thirdly, sampling was not random, although the sample size was large and obtained from different Spanish regions. This is why we also suggest caution when extrapolating these results and the study should be understood as an initial approach to the problem. We recommend adapting the OGD-Q to other regions and languages and replicating the evidence found. In addition, this tool should be linked to other diagnostic elements, such as interviews or complementary measures. That said, we also recommend exploring other processes that favor the diagnostic accuracy of this questionnaire, such as ROC (Receiver Operating Characteristic) curves.

Finally, according to our data, future lines of research might include the relationship between online video-game disorder and problematic online gambling including so-called loot boxes. These boxes, unlike direct purchases within a video game, involve investing a certain amount of money to buy the possibility of obtaining a reward (for example, a card, a character or material). Legally, loot boxes are not considered online gambling, but at a psychosocial level, they share the characteristics of gambling [56]. Further exploration of this association might reveal clinical and educational implications [57].

## 5. Conclusions

In conclusion, this work confirms the psychometric properties of the scores obtained on the OGD-Q. In addition, we obtained exploratory data on the prevalence of this growing problem, which are of special interest to pediatric and psychology units, as well as to those responsible for guidance counseling in schools. Parents can also benefit from this knowledge because education and parental supervision can play a very important role in the prevention of these problems associated with the Internet.

## Figures and Tables

**Table 1:** Result of the content validity of the Online Gambling Disorder Questionnaire (OGD-Q).

OGD-Q		DSM 5 Gambling Disorder 312.31 (F63.0)		ICD-11 6C50 Gambling Disorder 6C50.1 GD, Predominantly Online		Griffiths [20]
Item	Description	Criterion	Description	Symptom	Description	
1	Do you feel the need to spend more and more money to get the high you desire?	1	Need to bet ever-increasing amounts of money to achieve the desired excitement.			Tolerance
2	Do you feel nervous, irritated, or angry when trying to reduce or stop gambling online?	2	Are you nervous or irritated when you try to reduce or stop gambling?			Withdrawal symptoms
3	Have you tried to control, reduce or stop gambling online and have not been able to do so?	3	Have you made repeated efforts to control, reduce or stop gambling, always without succeeding?	1	Impaired control over gambling: onset, frequency, intensity, duration, termination, context.	Conflict: Intrapsychic conflict
4	Have you ever felt that online gambling has had negative consequences at a personal, social, family, or academic/work level, and you have still continued to gamble?	8	Have you endangered or lost an important relationship, a job, or an academic or professional career because of gambling?	3	Continuation or escalation of gambling despite the occurrence of negative consequences.	Conflict: interpersonal conflict
5	Do you often think about online gambling, for example, remembering past bets, planning your next bets, thinking about ways to make more money gambling online, reliving some moments related to online gambling, etc.?	4	Your mind is often full of betting (e.g., continually reliving in your imagination experiences of past bets, conditioning or planning your next bet, thinking of ways to get money to gamble).	2	Increasing priority given to gambling to the extent that gambling takes precedence over other life interests and daily activities.	Salience
6	Do you bet or gamble online when you feel sad, anxious, or guilty, in order to feel better or to stop thinking about how you feel?	5	You often gamble when you feel uneasiness (e.g., helplessness, guilt, anxiety, depression).			Mood modification
7	Do you feel like you have little control over online gambling (e.g., gambling more than you would like, spending more money than you would like, gambling in places where you shouldn’t do that, not being able to stop gambling when you want to)?			1	Impaired control over gambling: onset, frequency, intensity, duration, termination, context.	Conflict: Intrapsychic conflict
8	After losing money on a bet or in online gambling, do you usually gamble again to try to get that money back?	6	After losing money on bets, you usually go back another day to try to win (“chasing” your losses).			
9	Do you lie to others to conceal how much time you gamble or how much you actually spend on online gambling?	7	You lie to hide your degree of involvement in gambling.			
10	Have you ever asked someone for money to improve or overcome the bad economic situation that online gambling has caused you?	9	You count on others to give you money to relieve your desperate financial situation caused by gambling.			
11	Have you felt that you prioritized gambling over other areas of your life that had been more important before (e.g., studying, hanging out with friends, sleeping less if you gamble at night, etc.)?			2	Increasing priority given to gambling to the extent that gambling takes precedence over other life interests and daily activities.	Salience
12	If you have answered yes to any of the above questions, how long have you been feeling this way?		For diagnosis, the individual must present four (or more) of the criteria over a 12-month period.		Gambling behavior and other features are normally evident over a period of at least 12 months in order for a diagnosis to be assigned.	

**Table 2:** Means, standard deviations, item-total correlation, positive response percentage, and factorial loadings of the OGD-Q items (*n* = 883).

	M	SD	IT	%+	SFL	Skew.	Kurt.
Online Gambling Disorder Questionnaire (OGD-Q)							
1. Do you feel the need to spend more and more money to get the high you desire?	1.26	0.70	0.62	16.7	0.63	3.40	12.55
2. Do you feel nervous, irritated, or angry when trying to reduce or stop gambling online?	1.18	0.60	0.77	11	0.79	3.89	16.42
3. Have you tried to control, reduce, or stop gambling online and have not been able to do so?	1.23	0.70	0.71	12.4	0.74	3.61	13.40
4. Have you ever felt that online gambling has had negative consequences at a personal, social, family, or academic/work level, and you have still continued to gamble?	1.18	0.60	0.72	11.2	0.74	3.96	17.18
5. Do you often think about online gambling, for example, remembering past bets, planning your next bets, thinking about ways to make more money gambling online, reliving some moments related to online gambling, etc.?	1.19	0.61	0.79	11.3	0.81	3.79	15.24
6. Do you bet or gamble online when you feel sad, anxious, or guilty, in order to feel better or to stop thinking about how you feel?	1.20	0.67	0.79	10.6	0.81	3.87	15.40
7. Do you feel like you have little control over online gambling (e.g., gambling more than you would like, spending more money than you would like, gambling in places where you shouldn’t do that, not being able to stop gambling when you want to)?	1.21	0.68	0.84	10.9	0.86	3.80	14.86
8. After losing money on a bet or in online gambling, do you usually gamble again to try to get that money back?	1.26	0.76	0.72	13.6	0.73	3.32	10.78
9. Do you lie to others to conceal how much time you gamble or how much you actually spend on online gambling?	1.19	0.70	0.80	8.9	0.82	4.21	17.76
10. Have you ever asked someone for money to improve or overcome the bad economic situation that online gambling has caused you?	1.19	0.67	0.83	9.2	0.85	3.97	15.90
11. Have you felt that you prioritized gambling over other areas of your life that had previously been more important (e.g., studying, hanging out with friends, sleeping less if you gamble at night, etc.)?	1.20	0.67	0.80	10.3	0.82	3.86	15.21

Note: M = arithmetical mean; SD = standard deviation; IT = corrected item-total correlation; %+ = Percentage who responded positively (at least once); SFL = standardized factorial loadings; Skew = Skewness; Kurt = Kurtosis. For items in Spanish please see the supplementary Table S1.

**Table 3:** Total correlations between the risks in boys and girls according to the OGD-Q, the Internet Gaming Disorder Scale (IGD-20) dimensions, the GPIUS dimensions, and the total NMP-Q score.

	1.	2.	3.	4.	5.	6.	7.	8.	9.	10.	11.	12.	13.	α	ω
Online Gambling Disorder	-	0.19 **	0.17 *	0.20 **	0.26 **	0.28 **	0.18 **	-0.03	0.13 *	0.12	0.10	0.08	0.05	0.94	0.95
Prominence (IGD-20)	0.16 **	-	0.37 **	0.73 **	0.69 **	0.54 **	0.64 **	0.23 **	0.21 **	0.17 *	0.26 **	0.33 **	0.20 **	0.72	0.73
Mood Mod. (IGD-20)	0.09 *	0.52 **	-	0.49 **	0.47 **	0.36 **	0.34 **	0.14 *	0.21 **	0.11	0.10	0.08	0.05	0.70	0.72
Tolerance (IGD-20)	0.19 **	0.76 **	0.50 **	-	0.77 **	0.52 **	0.67 **	0.23 **	0.18 **	0.25 **	0.32 **	0.32 **	0.23 **	0.74	0.78
Abstinence (IGD-20)	0.23 **	0.72 **	0.46 **	0.76 **	-	0.61 **	0.59 **	0.24 **	0.23 **	0.29 **	0.32 **	0.37 **	0.24 *	0.82	0.82
Conflict (IGD-20)	0.23 **	0.61 **	0.36 **	0.60 **	0.67 **	-	0.46 **	0.22 **	0.07	0.22 **	0.24 **	0.36 **	0.12	0.70	0.72
Relapse (IGD-20)	0.20 **	0.67 **	0.44 **	0.65 **	0.70 **	0.56 **	-	0.14*	0.10	0.20 **	0.33 **	0.28 **	0.21 **	0.71	0.72
Pref. Onl. Soc. Int. (GPIUS2)	0.05	0.26 **	0.28 **	0.27 **	0.28 **	0.22 **	0.27 **	-	0.44 **	0.45 **	0.44 **	0.40 **	0.26 **	0.79	0.80
Mood Reg. (GPIUS2)	0.03	0.25 **	0.44 **	0.24 **	0.26 **	0.18 **	0.22 **	0.48 **	-	0.49 **	0.44 **	0.50 **	0.36 **	0.83	0.84
Neg. Cons. (GPIUS2)	0.09 *	0.36 **	0.34 **	0.36 **	0.42 **	0.35 **	0.36 **	0.49 **	0.49 **	-	0.78 **	0.55 **	0.55 **	0.77	0.79
Cog. Concern (GPIUS2)	0.04	0.35 **	0.29 **	0.36 **	0.36 **	0.32 **	0.36 **	0.42 **	0.43 **	0.73 **	-	0.62 **	0.57 **	0.89	0.89
Compulsive Use (GPIUS2)	0.10 *	0.39 **	0.39 **	0.35 **	0.39 **	0.27 **	0.35 **	0.30 **	0.37 **	0.53 **	0.39 **	-	0.36 **	0.86	0.88
Nomophobia	0.16 **	0.52 **	0.50 **	0.76 **	0.67 **	0.27 **	0.35 **	0.30 **	0.37 **	0.53 **	0.39 **	0.41 **	-	0.95	0.96

Note: Mood Mod. = Mood modification. Pref. Onl. Soc. Int. = Preference for Online Social Interaction. Mood Reg. = Mood Regulation; Neg. Cons. = Negative Consequences. Cog. Concern = Cognitive Concern. Reliability indices (Cronbach alpha α and omega coefficient ω) are shown in the last two columns. *N* = 843; boys (*n* = 602); girls (*n* = 241). Boys’ correlations are below the diagonal girls’ are above it. ** *p* < 0.001. * *p* < 0.05.

**Table 4:** Total correlations between the OGD-Q and the three dimensions of DASS-21 (Depression, Stress, and Anxiety) for boys (*n* = 602) and girls (*n* = 241).

	1.	2.	3.	4.	α	ω
Online Gambling Disorder	-	0.16 *	0.21 *	0.17 *	0.94	0.95
Stress (DASS-21)	0.19 **	-	0.86 **	0.82 **	0.92	0.92
Anxiety (DASS-21)	0.29 **	0.83 **	-	0.79 **	0.87	0.87
Depression (DASS-21)	0.25 **	0.81 **	0.82 **	-	0.89	0.89

Note: The reliability indices (Cronbach alpha α and omega coefficient ώ) of the dimensions are shown in the last two columns. Boys’ correlations are below the diagonal girls’ are above it. ** *p* < 0.001. * *p* < 0.05.

**Table 5:** Relationship between the number of adolescents who score as having a “problem” in n-items of the OGD-Q and their prevalence compared to the total number of participants and the total number of participants who have sometime gambled online.

	For More than 12 Months			Between 6 and 12 Months			Between 1 and 6 Months			Recently		
*N* of Indicators	*n*	%T	%G	*n*	%T	%G	*n*	%T	%G	*n*	%T	%G
1	18	0.67	2.04	7	0.26	0.79	7	0.26	0.79	19	0.71	2.15
2	2	0.07	0.23	4	0.15	0.45	1	0.04	0.11	3	0.11	0.34
3	2	0.07	0.23	1	0.04	0.11	0	0.00	0.00	5	0.19	0.57
4	6	0.22	0.68	2	0.07	0.23	2	0.07	0.23	1	0.04	0.11
5	4	0.15	0.45	2	0.07	0.23	1	0.04	0.11	3	0.11	0.34
6	4	0.15	0.45	3	0.11	0.34	0	0.00	0.00	0	0.00	0.00
7	1	0.04	0.11	7	0.26	0.79	1	0.04	0.11	1	0.04	0.11
8	0	0.00	0.00	1	0.04	0.11	1	0.04	0.11	0	0.00	0.00
9	0	0.00	0.00	3	0.11	0.34	0	0.00	0.00	0	0.00	0.00
10	6	0.22	0.68	2	0.07	0.23	0	0.00	0.00	3	0.11	0.34
11	3	0.11	0.34	1	0.04	0.11	2	0.07	0.23	0	0.00	0.00
*n* (≥ 4 sympt.)	24	0.89	2.71	21	0.77	2.38	7	0.26	0.79	8	0.3	0.90

*n*: number of subjects; %T: percentage of total participants (*n* = 2691); %G: percentage of total participants who have gambled sometime (*n* = 883); *n* (≥ 4 sympt.) = number of participants with 4 or more criteria/items with problems.

**Table 6:** Descriptive statistics, significance and effect size of differences between participants with and without online gambling disorder in all three dimensions of the DASS-21.

Instrument	*n* (< 4 sympt.)	M ± SD (< 4 sympt.)	*n* (≥ 4 sympt. and 12 Months or More)	M ± SD (≥ 4 sympt. and 12 Months or More)	*t*-Welch (*p*)	Effect Size (Cohen’s *d*)
DASS-21 (Stress)	810	11.53 ± 4.83	22	14.86 ± 6.81	−2.277 (.033)	0.68
DASS-21 (Depression)	797	10.74 ± 4.87	20	14.75 ± 7.38	−2.149 (.026)	0.81
DASS-21 (Anxiety)	800	10.53 ± 4.37	22	14.40 ± 6.48	−2.782 (.011)	0.87

Note: *n* (< 4 sympt.): participants with fewer than four criteria/items with problems; *n* (≥ 4 sympt.): participants with four or more criteria/items with problems in the past 12 months or longer. M = arithmetical mean; SD = Standard deviation.

## Supplementary Materials

The following are available online at https://www.mdpi.com/2077-0383/9/1/120/s1, Table S1: Cuestionario Diagnóstico del Juego de Azar Online.
