# Supplementary material for: Design and Measurement Properties of the Online Gambling Disorder Questionnaire (OGD-Q) in Spanish Adolescents
Source: J Clin Med. 2020 Jan 2;9(1):120. doi: 10.3390/jcm9010120 (PMC7020023; doi:10.3390/jcm9010120)
Supplement: Supplementary file 1 [file jcm-09-00120-s001.pdf]

Cuestionario Diagnóstico del Juego de Azar Online

(OGD-Q; González-Cabrera, Machimbarrena, Beranuy, Pérez-Rodríguez, Fernández-Gonzáles y Calvete, 2020)

En el siguiente cuestionario encontrarás afirmaciones relacionadas con el juego de azar online. Esto hace referencia a todos los juegos online en los que participa la suerte, por ejemplo, partidas de póker online, bingos online, casas de apuestas online e incluso la compra de sobres, cajas o cofres en videojuegos (FIFA, Hearthstone: Heroes of Warcraft, CS-GO, etc.). Cualquier gasto de dinero para comprar una apuesta donde no sabes si te tocará o no el premio deseado puedes considerarlo un juego de azar online. Teniendo eso en mente, responde a estas preguntas, por favor:

	Nunca	*En alguna ocasión puntual*	*Con frecuencia*	*Muy frecuente-mente*	*Todos los días*
1. ¿Sientes la necesidad de gastar cada vez más dinero para conseguir el subidón que deseas?	1	2	3	4	5
2. ¿Te sientes nervioso, irritado o enfadado cuando intentas reducir o dejar el juego de azar online?	1	2	3	4	5
3. ¿Has intentado controlar, reducir o abandonar el juego de azar online y no has podido hacerlo?	1	2	3	4	5
4. ¿Has sentido alguna vez que el juego de azar online ha tenido consecuencias negativas a nivel personal, social, familiar o académico/laboral, y aun así has seguido jugando?	1	2	3	4	5
5. ¿Piensas a menudo en las apuestas online, por ejemplo, recordando apuestas pasadas, planificando tus próximas apuestas, pensando en formas de ganar más dinero jugando online, reviviendo algunos momentos relacionados con el juego de azar online, etc.?	1	2	3	4	5
6. ¿Apuestas o juegas a juegos de azar online cuando te encuentras triste, ansioso o te sientes culpable, para sentirte mejor o dejar de pensar en cómo te sientes?	1	2	3	4	5
7. ¿Sientes que tienes poco control sobre el juego de azar online (p. ej., jugar más de lo que te gustaría, gastar más dinero de lo que quisieras, jugar en sitios en los que no deberías hacer eso, no poder parar de jugar cuando quieres…)?	1	2	3	4	5
8. Después de perder dinero en una apuesta o en un juego de azar online, ¿sueles volver a jugar para intentar recuperar ese dinero?	1	2	3	4	5
9. ¿Mientes a los demás para ocultar cuanto tiempo juegas o cuanto gastas realmente en juegos de azar online?	1	2	3	4	5
10. ¿Has pedido dinero a alguien para mejorar o superar la mala situación económica que te ha causado el juego de azar online?.	1	2	3	4	5
11. ¿Has sentido que dabas prioridad al juego de azar por encima de otras áreas de tu vida que antes habían sido más importantes (p. ej., estudiar, salir con los amigos, dormir menos si juegas de noche, etc.)?	1	2	3	4	5

12. Si has marcado que has sentido algunas de las situaciones anteriores (sientes la necesidad de gastar más dinero, te sientes mal al dejar de jugar, sientes que jugar tiene consecuencias negativas, piensas a menudo en las apuestas online, sientes que tienes poco control sobre el juego, sigues apostando aun habiendo perdido dinero, etc). **¿Desde cuándo sientes esto?**

- Hace más de doce meses
- Hace más de seis meses
- Hace más de un mes
- Recientemente
